# Facial Neuralgia, from a Dento-Surgical Standpoint

**Published:** 1895-03

**Authors:** M. H. Cryer

**Affiliations:** Philadelphia, PA.


ARTICLE II.

### FACIAL NEURALGIA, FROM A DENTO-SURGICAL STANDPOINT.

BY M. H. CRYER, M. D., D. D. S., PHILADELPHIA, PA.

Read before the New Jersey State Dental Society, July 19, 1894.

That there are few diseases more baffling to the practitioner than facial neuralgia, is shown by the many and differing opinions concerning its diagnosis and treatment. When, however, the cause is apparent, and can be located in optical defects, diseased teeth, etc., the neuralgia is called peripheral, and, properly combated, yields readily to local treatment. But if the cause be obscure, and cannot be traced to any peripheral disturbance, a diagnosis is difficult. A number of surgical operations, such as resection of a branch of the fifth nerve, either within or without the brain case, removing and destroying the Gasserian ganglion, or cutting the sensory nerve beyond the ganglion, are practiced. Medical treatment is only effective when the neuralgia is idiopathic, or arises from some constitutional cause. The continued administration of sedatives and narcotics usually leads to the habitual use of this class of drugs.

The fifth pair of nerves being the ones almost exclusively associated with this affection, a *resume* of the local anatomy is advisable. A clearer understanding of the aberrations of functions which we are to study in connection with this nerve can, I think, be had by inverting the usual order of descriptive study of its anatomical distribution by beginning our investigation from the standpoint of its peripheral terminations.

The peripheral end-organs of the fifth nerve, found, with few exceptions, in all the tissues of the face, mouth, jaws, nasal chambers, and orbits, are receivers of impressions. As the outer sentinels to this region, they are of the utmost importance to the general economy. It is necessary that they shall be of a character of great sensibility and watchfulness.

The nerves proper are white fibrous cords of various sizes, connecting peripheral end organs with the sensorium, and also one nerve-center with another. They do not generate nerve-force, but act as conductors or conveyors of impressions. Nerve-fibers vary in size from 1-2,000 to 1-1,200 of an inch in diameter. Each, in section, consists of an axis-cylinder, medullary sheath, and neurilemma. The axis-cylinder is the essential portion of the nerve-fiber, being nearly uniform in diameter, and undergoing no interruption or bifurcation from near its peripheral end-organs to the nerve-center. The medullary sheath and the neurilemma with their interspaces are believed to act in a great measure as insulators and protectors to the axis-cylinder.

The ganglia belonging to and closely associated with this nerve are five in number,—the Gasserian, ophthalmic, spheno-palatine, sub-maxillary, and otic. The Gasserian ganglion rests in a depression on the superior part of the anterior surface of the apex of the petrous portion of the temporal bone. It is broad, flattened and somewhat crescent shaped, and it receives on the inner side filaments of communications from the carotid plexus and a few branches between the dura mater and the tentorium. The ophthalmic, spheno-palatine, submaxillary, and otic ganglia are situated in the regions indicated by their respective names. They properly belong to the sympathetic system of nerves, and, as in all ganglia of this class, they have three sets of fibers, communicating with different divisions of the fifth nerve, and with branches from motor nerves. It is through these ganglia that many sympathetic or reflex nervous phenomena are accounted for. The sensorium of the nervous system is situated within the brain-case, and in it must be the common center of sensation.

The ultimate number of sensitive nerve-filaments of the fifth pair is estimated on either side at thirty thousand. Beginning at the periphery, they converge and unite into bundles, which are covered by an envelope. These bundles form branches, which in turn unite, making three great divisions, named the ophthalmic, the superior maxillary, and the sensitive portion of the inferior maxillary. These as trunks enter the brain-case respectively through the anterior lacerated foramen, the round and the oval foramina. All three perforate the sphenoid bone. After passing into the cranium, they enter the Gasserian ganglion at its anterior aspect. Issuing from the ganglion, they are united into what is known as the posterior or sensory root of the nerve. This extends backward through the pons Varolii to the floor of the fourth ventricle in the medulla oblongata.

The reception of impressions, whether of sight, sound, taste, smell, or touch, is caused by irritation of the special end-organs of sensory nerves. The sensation is carried by the nerve to the sensorium; for example, the sense of right. The end-organs of the optic nerve are found in the retina. As the various types of light pass through the eye, they strike the retina and cause stimulation of the rods and cones. The effect is in accord with the amount and quality admitted. Thus received, it is conveyed through the nerve to the centers of vision.

I once heard a well-known physician ask a celebrated professor of neurology, after he had delivered a lecture, "What is neuralgia? What is pain? What is it that feels?" It created a most interesting discussion, but the questions are still unanswered, and remain a problem referable to the special domain of physiological psychology.

Can we say positively where the true line of demarkation is, between a pleasurable sensation and severe pain.

I think not. Of course, at the extreme of each senation there is decidedly an intrinsic pain or pleasure; but where is the line to be drawn? As each man has a different standard of happiness, so must his ideas of pain or pleasure vary. The simple pleasure of the laborer would be exceedingly tiresome to the man of more refined taste.

Satiety always produces disgust; pleasure, if carried to excess, becomes unendurable. Suppose one listens to a beautiful strain of music, have this same strain continued without variation for a length of time, and it will become monotonous and even painful. It is the same with taste and other sensations.

From the dento-surgical standpoint, facial neuralgia is recognized as being caused by undue irritation or disease of the peripheral end-organs; by injury, compression, or disease of the nerve-fibers between the periphery and the sensorium; by reflex or sympathetic action; by disease or compression; and finally by disorder of the general system.

In order to better illustrate nerve-irritation, we will examine the general workings of an electrical bell circuit, comparing its action with a corresponding action in the nervous system. The battery will represent the force-generator or the nerve-center; the push-button, end-organs; the insulated wires, nerves; and the indicator, the motor periphery. By pushing the button the wires convey the impression to the indicator, which will tell what button is to be answered. Now, if we look at the nervous system in the same way, the end-organs, being irritated, convey that impression to the nerves, which carry it to the sensorium, and we have something like sixty thousand push-buttons, nerves, and receivers of impressions in the system of the fifth pair of nerves. If by some accident or disease the button or end organ become disarranged, there would either be no response or we might have a constant irritation, according to the nature of the disturbance; but if the parts are in normal condition, the sensation will cease at the moment the pressure or the irritation is removed.

Should the nerve itself become diseased, as in neuritis or from the formation of a neuroma, or be in any way unduly compressed, so as to overcome the effects of the insulation of the axis-cylinder, pain will be felt referable to the end organs. A common example of this is the striking of the ulnar nerve against the elbow-joint, vulgarly known as the "crazy-bone." Pain will be felt in the hand at the parts of the end-organs belonging to the nerve-fibers compressed. In taking the wires of this system before you, and compressing them sufficiently to overcome the insulating properties, we will find that the indicator rings and denotes by its letter that either the button or the wire belonging to it has been disturbed. The electrician would examine the push-button and wires, and on finding the trouble, repair the injury. If he have not the power to do this, a more experienced one is called for aid. The dentist or surgeon holds the same position in relation to the human body in case of neuralgia. If he have not the ability to make a complete diagnosis from the end-organs to the sensorium, either by direct examination or by inductive reasoning, he should call to his aid the best assistance obtainable.

The diagnosis of facial neuralgia and locating the cause are of great importance, and among the most difficult problems the surgeon has to solve. Diagnosis by exclusion, or in other words ascertaining what a thing is by eliminating what it is not, is the best method. As neuralgia may be confounded with other diseases, each of these must first be excluded. For instance, the possibility of its being a case of neuritis. In determining the nature of this case, all signs of inflammation must be considered, and if not found, that disease can generally be excluded. After convincing oneself that the case is one of neuralgia, determining the cause is the next step, and here also the process of exclusion should be followed. It is my opinion that the peripheries of the fifth nerve should be exhaustively examined. The care of the mouth should be in the charge of a competent dentist, and I mean by this word all that is properly included in the term stomatologist; each tooth examined for hypersensitive dentine, superficial and deep decay, exposed pulps, fillings that are irritating to the pulp, pulps in any stage of inflammation, whether they be in sound or decayed teeth, or in one that has been filled. The remains of roots, and whether a fistula opens to them or the gum-tissue has grown over them; normal and even supernumerary teeth that have not been erupted must be determined. I present for your inspection three teeth, a cuspid, central, and a lateral incisor, which were found on opening an antrum in search for a cause of neuralgia; the three perfect crowns were protruding into the antrum, while the imperfect roots were imbedded in the anterior portion of the inner walls. After their extraction the neuralgia ceased. Sometimes teeth in apparently healthy condition may be dead and have caused abscesses that are no longer connected with them, some of which may have opened into the antrum, the pus burrowing under the muco-periosteum of that cavity, elevating it until it closes the opening between the antrum and nasal cavity. This is one source of neuralgia. Cicatricial tissue must also be looked upon with suspicion. In fact, the mouth and its surroundings must be put in such condition that it can be excluded as the cause of the disease under consideration.

An examination should be made over the entire region of the branches of the nerves. It should be ascertained if the patient has at any time received injury of any kind in or about the head or face. This causes neuralgia not infrequently by having some of the nerve-filaments caught in a cicatrix, or they may be compressed against bony structure. Failing to localize an injury) the constitutional diseases should next be surveyed,– gout, anæmia, rheumatism, malaria, syphilis, catarrh, etc., any and all of which cause neuralgia. Gout and rheumatism, however, seldom produce neuralgia of the head; but malaria, syphilis, and catarrh are common causes. If the patient is suffering from any of these complaints, it should be treated and cured as a step in the diagnosis.

Not until all external and costitutional causes of neuralgia have been excluded is the surgeon justified in opening the face or brain-case for a resection or the removal of the Gasserian ganglion. A tendency of the general surgeon of to day is to disregard the importance of thorough examination and correction of the discoverable diseases, especially those of the mouth, and he may, in consequence, perform an extensive operation regardless of disfigurement or the risk of losing his patient.

Dr. Edward C. Kirk relates the following case which shows such an oversight or ignorance. A patient suffering from neuralgia was sent to him by a well-known rhinologist and larynologist to have the mouth carefully examined for an etiological factor in a neuralgia. Dr. Kirk found three lesions, any one of which could have caused the whole trouble. He made a report to the specalist, and arrangements were made to have him remove the cause of local irritation within the mouth. While awaiting the time for operation the patient was seen by his family physician, who strongly and successfully urged him to call a general surgeon, who at once disapproved of the slight operations within the mouth. He advised and obtained consent of the patient to an operation for resection of a nerve-trunk. I have not the least doubt that there is not a member of this organization who has not relieved what appeared to be an obscure case of neuralgia by finding and curing some lesions of the mouth. The eye, ear, and nose specialists have had similar experience, all of which proves that the peripheral causes of neuralgia should receive the closest attention before searching for the obscure causes. In many instances a patient goes from one specialist to another, receiving possibly temporary benefit from each, but sooner or later the old pain returns, and there is no peripheral cause left upon which to speculate; it is then that difficult and delicate surgery is necessary.

The two cases to be detailed later are illustrations that the surgical operations of resection should be the last resort. In the first case, that of Mr. B., it was not until the eleventh operation that the true lesion was found in neuromata, but these growths before operation gave no indication of their presence.

After an experience of fifteen years in the special studies of diseases and surgery of the mouth, face, and neck, I have become more thoroughly convinced each year that it will be the properly trained scientific dentist who will not only cure facial neuralgia, but to a great extent prevent it by judicious work. The entire nervous system is a highly organized complexus of elements; less understood than the other great anatomical divisions. Nerves being nourished, like other tissue, through the vascular system, are subject to inflammation. When a portion of their structure is called upon for action, more blood is required in the parts, the nerves, especially the nerve endings, are stimulated, and the tissues surrounding them become hyperemic. This may be seen when an inflamed pulp is punctured; there will be, in addition to the vascular, greater change in the nerve-tissue. Like other tissue, if unduly overworked, disease will result; in some cases hypertrophy, in others atrophy. The nerves of the teeth pass through foramina and canals within hard bony structures; if they increase in size they will become impinged upon, the irritation causing an excess of blood in the parts. The lesson to learn from this is that any operation should give as little pain as possible, and after its completion there must be nothing left to cause further irritation, whether it be a bridge, a pulp capping, or a filling placed in such a position or of such a character as will cause pulp irritation. We are so accustomed to find pain passing away in a few days that we often forget that all patients are not alike.

There are two cases to which I wish to draw your attention. The first I reported to the World's Columbian Dental Congress, and think it of enough interest to repeat, especially as another sixteen months have passed away with no return of pain. Mr. B. had been suffering for many years, and after the possibility of any peripheral trouble was excluded, had had eight distinct and separate operations performed by some of the most skillful surgeons for the relief of facial neuralgia in the inferior maxilla. On October 8, 1892, he came before the clinic of the Hospital of Oral Surgery, and the following operation was performed. The lower lip was divided at the median line to the lower border of the mental process, and an incision made on the line of the bone to the insertion of the masseter muscle. The lip and cheek were then dissected from the bone, and the inferior dental canal opened by suitable burs driven by the surgical engine. The nerve was cut from the canal and the soft parts readjusted. On recovery from the effects of the anesthetic, the patient felt no relief. The seat of the pain seemed to be in the body of the bone, especially on the inner side, so that on the following Saturday, October 15, it was thought advisable to reopen the the parts, and by the use of the engine bur the greater portion of the body of the bone of the left side was then removed, but no relief was obtained. Time was given for the parts to heal and the patient to recover from the shock, also in the hope that the pain might pass away, but it became, if possible, worse. The patient's sufferings were so intense that he would throw himself on the floor and cry for relief. Finally it was decided to perform an operation which I had suggested, and which was first performed at the clinic of the Medico-Chirurgical Hospital February 14, 1891. After deepening the sigmoid notch by the use of a barrel-shaped engine bur, the inferior maxillary nerve was fully exposed. The patient, well under the influence of ether, did not respond to the cutting of the bone or other tissue, but as soon as the nerve was seized by hemostatic forceps he exhibited by his movements and outcry the agony of pain. The nerve was drawn out of the inferior dental canal and cut off as high and near the oval foramen as possible. Upon the section of the nerve removed there were two large and distinct neuromata, the lower one being impinged upon by the triangular process of bone at the opening of the inferior dental canal. The upper one lay under the external pterygold muscle. Beyond a doubt, here was the cause of the present neuralgia. It would be an interesting question to decide if it were the original cause. I have doubts of this, however, and think it more likely that the trouble had its incipiency in or about the jaw, which causing peripheral irritation produced vascular and nervous excitement, it being carried to such an extent that eventually the nerve became diseased at the entrance to the inferior dental canal. The patient came from under the influence of ether without the slightest pain, and has not had a twinge up to that time. This case is illustrative of a lesion in the course of the nerve, although the sensorium indicated it to be in the lower jaw, the latter being the seat of the peripheral end-organs of the nerve-filaments affected by the neuromata. If the patient had received no benefit from this operation, the next would have been the removal of the Gasserian ganglion.

The other case is one of greater interest to us, not only from a dento-surgical standpoint, but that the patient is a brother practitioner of dentistry. For me he holds other bonds of union, as he was formerly a student of mine. I have assisted in performing nine extensive operations in and about his head, face and jaws, the last one being the removal of the Gasserian ganglion. His case of neuralgia has been one of almost constant study for thirteen years, upon which chapters could be written, but time will allow but a brief mention. Those who are further interested in the case can find an account of it in detail in the Transactions of the Philadelphia County Medical Society for 1894, in which will be found an admirable report by the gentleman whe removed or destroyed the Gasserian ganglion so successfully, Professor W. W. Keen, M. D., of Philadelphia. There is also an extract of this report to be found in the *International Medical Magazine* for May, 1894, to which I am indebted for the greater portion of the following:

J. T. K., male, aged forty-two years. Until his thirtieth year was in excellent health, and had no indications of any tendency to neuralgic disease.

In 1880, without any previous warning, he had a sudden attack of violent pain in the right upper jaw, nearly limited to one tooth. This came on during a meal. The tooth was extracted, but the pain continued in the alveolus from which the tooth was drawn, until it healed, when it transferred itself to another tooth, and the process was repeated until three were extracted. But one of these, the second molar, which had been filled some years before with tin and amalgam, was found in an unhealthy condition.

The next operation consisted in cutting loose the gum from the bone for the whole depth of tho process, both on the palatal and buccal sides, as it was supposed by Dr. Levis that the pain was caused by the gums contracting so as to press tightly upon the nerve radicles. This operation gave no relief.

After suffering agony for some weeks, the upper teeth were extracted, one cuspid being broken and giving rise to necrosis; the root and a portion of necrosed bone were removed afterward by Professor Garretson.

For a short time the pain in the face was relieved, but became finally so severe in the region of the infraorbital foramen that Professor Garretson cut down upon the infraorbital nerve and removed all the portion anterior in the foramen. This was in September, 1881.

In the spring of 1883 pain returned in a small era in the side of the upper jaw, and the sixth operation was undertaken by Professor Garretson, a portion of the upper jaw being removed, exposing the antrum. The patient had comparative ease until the fall of that year, when the pain returned, and he was treated during the succeeding four or five years without much relief.

In the winter of 1887-88, he suffered as only neuralgics do. Professor Garretson made a resection of the second division of the fifth nerve near the foramen rotundum.

In March, 1889, the third division of the fifth nerve was resected at the foramen ovale by the same surgeon. This gave much relief, but the pain soon returned, and the ninth operation, the ligation of the facial artery, was done in 1890. In September of the same year, the tenth operation, an excision of nearly all the superior maxilla, took place. In September, 1891, Professor Garretson did the eleventh operation, opening, at the patient's request, the inferior dental canal, and removing the nerve as far forward as the mental foramen.

From most of the operations the patient experienced some temporary relief. On admission to the Orthopædic Hospital, in May, 1893, he was suffering from three or four paroxysms of severe pain a day, for the relief of which he was taking half a grain of morphine twice a day, and more if the attack was too painful to bear.

The removal of the Gasserian ganglion was proposed by Dr. Keen, but as the patient preferred that another operation upon the inferior dental nerve should be tried first, Dr. Keen consented to this, ahd on June 10, 1893, that operation was performed.

The operation not giving relief, on the 28th of August Dr. Taylor removed the tissues about the infraorbital foramen, but with only temporary relief, the pain returning, and not being relieved by even large and frequently repeated doses of morphin.

On October 18, 1893, Dr. Keen made an omega-shaped incision, the length of which, vertically, was three inches. One leg terminated immediately in front of the tragus, the other just in front of the anterior and middle third of the distance between the external auditory meatus and the external angular process. The temporary artery was cut, and this and a few other vessels required ligation. Dr. M. H. Cryer, with the new surgical engine of The S. S. White Company, using a circular saw of one and a half inches in diameter, with a guard, then rapidly and very successfully divided the external table, excepting at the two extremities. Here, fearing perforation before the inner table and the rest of the area were divided, Dr. Keen chiseled through the superior four fifths of the section, passing through the inner table with ease, and then chiseled the extremities. At the posterior terminus, when giving but a light blow with the mallet, the chisel suddenly penetrated the skull to a depth of perhaps half an inch, and immediately a considerable hemorrhage occurred, showing that the posterior branch of the middle meningeal probably had been cut.

Dr. Keene said, "I then chiseled quickly through the anterior extremity, broke the bone, and reflected the flap. Here again was an additional trouble, for the anterior branch went through a distinct canal at the anterior inferior angle of the parietal bone, and of course was torn and bled freely as I turned down the flap of bone and scalp. A finger over each artery, however, almost controlled the hemorrhage, and in a very few moments I was able to place a pair of hemostatic forceps on each. A cut was seen in the dura corresponding in length to the width of my chisel. A small, curved Hagedorn needle was then passed first around the anterior branch of the dura, then the posterior branch was caught. The opening in the dura was sutured by catgut. The middle lobe of the brain was then lifted with care and great gentleness from the bone, but this was followed by hemorrhage which might well be called alarming. Lifting up the middle lobe with a spatula, I discovered that it was not as I had feared, from the middle meningeal ruptured at the foramen spinosum, but that the blood came from the neighborhood of the ganglion. I could not think, with the gentleness I had used, that the cavernous sinus had been ruptured, but at all events the flow was too free not only to not allow of removal of the ganglion, but the man's life would be jeopardized. The hemorrhage did not yield to hot water. I packed the wound twice with gauze, which arrested the bleeding temporarily, but on removing the gauze hemorrhage was as profuse as before. Therefore I packed in a considerable piece of iodoform gauze and closed the flap by six retention stitches, with the intention of removing the packing in two or three days, and then removing the ganglion."

Without any marked symptoms occurring, the wound, which was partly united, was re-opened on the third day and the gauze carefully removed, a stream of hot water being used to loosen it.

The hemorrhage, which was slight, was readily controlled by small hot sponges. The cavernous sinus and the middle meningeal artery were both found intact. The second and third division of the fifth nerve, and also the flap of the dura which covered the ganglion, were soon found, though great trouble was experienced in separating the third division from the middle meningeal, as they were very close together. Moreover, both at the second and third divisions, the slightest touch of the blunt hook, Allis's dissector, scissors, knife, anything produced the sudden escape of at least a drachm of blood, which obscured the field of operations. Pressure by a small gauze sponge for a very few moments would cause the bleeding to cease, but the slightest manipulation produced its recurrence. However, after cutting both of these nerves and tracing them back to the ganglion, the little extradural sac was torn open, and with the Allis dissector and a small sharp spoon the ganglion was thoroughly destroyed.

The wound was then closed and the usual dressings applied. The patient's condition, both throughout the operation and at its end, was decidedly better than in the operation of the 18th.

He made a complete and rapid operative recovery, and was out of bed on the fourth day, the entire wound being healed. The highest temperature was 101.2° F.

At first it was feared that permanent injury would follow, as he suffered from delusions, aphasia, and other symptoms, but fortunately these all passed quickly away.

The engine used in this case, as described by Dr. Cryer, is comparatively a new one, having been made during the last summer; it embodies suggestions of his own in regard to giving great power, speed, and steadiness, the hand-piece has also been arranged so that different guards and cutting instruments can be readily and securely placed, the engines which have been heretofore used lacking these qualities.

Of the saw and guard used in making the initial section of the brain-case for the operation above described, Dr. Cryer said, "The guard is set by the thumb-screws, fixing the depth the saw shall cut. In this case it was the intention to cut through the outer table and diploe, but not through the inner plate; this was successfully accomplished and with rapidity. The inner table was broken by Dr. Keen, using mallet and chisel. This operation, as well as carefully conducted experiments on the cadaver, demonstrated that this instrument was not all that was wanted for opening the brain-case, the difficulty being that skulls vary in thickness, not only in different subjects, but in individual cases. The cutting instrument should be so devised that both tables can be cut without danger to the dura, regardless of the thickness of the bone and without the subsequent use of mallet and chisel. In order to overcome this objection, I have devised the spiral osteotome and guard, for a description of which see *Dental Cosmos*, January, 1894, and November, 1894."

The following is from a foot-note in Professor Keen's report of the "J. T. K." case, recited above: "In a later case at the Jefferson Hospital Dr. Cryer kindly used this instrument (the spiral osteotome) for me with great satisfaction."—*Dental Cosmos.*

